# Hybridized bio-inspired intrusion detection system for Internet of Things

**DOI:** 10.3389/fdata.2023.1081466

**Published:** 2023-02-01

**Authors:** Richa Singh, R. L. Ujjwal

**Affiliations:** University School of Information, Communication, and Technology, Guru Gobind Singh Indraprastha University, Dwarka, New Delhi, India

**Keywords:** Internet of Things, intrusion detection system, salp swarm algorithm, sine cosine algorithm, feature selection

## Abstract

The Internet of Things (IoT) consists of several smart devices equipped with computing, sensing, and network capabilities, which enable them to collect and exchange heterogeneous data wirelessly. The increasing usage of IoT devices in daily activities increases the security needs of IoT systems. These IoT devices are an easy target for intruders to perform malicious activities and make the underlying network corrupt. Hence, this paper proposes a hybridized bio-inspired-based intrusion detection system (IDS) for the IoT framework. The hybridized sine-cosine algorithm (SCA) and salp swarm algorithm (SSA) determines the essential features of the network traffic. Selected features are passed to a machine learning (ML) classifier for the detection and classification of intrusive traffic. The IoT network intrusion dataset determines the performance of the proposed system in a python environment. The proposed hybridized system achieves maximum accuracy of 84.75% with minimum selected features i.e., 8 and takes minimum time of 96.42 s in detecting intrusion for the IoT network. The proposed system's effectiveness is shown by comparing it with other similar approaches for performing multiclass classification.

## 1. Introduction

IoT (Hussain et al., 2020) consist of several interconnecting devices known as things, with limited communication, computation, and storage capability. These devices can vary from simple household devices such as smart bulbs, smart fridges, smart meters, IP cameras, etc. to more complicated devices such as RFID, smart devices used in industry, heartbeat detectors, etc. IoT system (Al-Fuqaha et al., 2015) is gaining importance in every field including smart grid, smart agriculture, smart transportation, smart home, semantic web, etc. The exponential growth of IoT devices gives a platform to intruders to perform malicious activities. The intruder aims to exploit IoT resources by launching cyber-attacks against IoT devices and networks, which is destructive. An IoT system is susceptible to various types of threats (Chaabouni et al., 2019) such as man-in-middle attacks, denial of service, routing attacks, eavesdropping, etc. As a consequence, the IoT system security is of prime importance. An intrusion detection system (IDS) is one of the security methods for the IoT system, which aims to detect and report any breach. Based on the detection strategy (Khraisat and Alazab, 2021), the IDS is classified as signature IDS, anomaly IDS, and hybrid. In signature IDS (Thakkar and Lohiya, 2021), network traffic is examined for the attack signatures stored in the database, any match is considered as malicious. In anomaly IDS, the user profile is created based on analyzing the network usage. Any variance from the usage behavior is considered an anomaly. Hybrid IDS merges the advantages of signature IDS and anomaly IDS. The data produced by the IoT network is voluminous plus heterogeneous. Therefore, having an effective and efficient feature selection (FS) method is important. The FS method selects the best features from the original set based on certain criteria. The FS methods are classified as, filter, wrapper, and hybrid (Balasaraswathi et al., 2017). In the filter method, various statistical measures such as correlation, information measure, and distance are used for selecting the feature subset. However, such methods don't interact with the classifier for the evaluation of feature subsets. The wrapper method interacts with the classifier for the evaluation of the selected feature subset. The hybrid method combines the merits of the filter and wrapper method.

Many works were published where metaheuristic algorithms (MHA) are used for the feature selection tasks of IDS. For instance, Kareem et al. (2022) proposed an efficient feature selection algorithm for IoT-based IDS. They hybridize the bird swarm algorithm (BSA) with the gorilla troops optimization (GTO) algorithm for the feature selection task. The exploration part of gorilla troops optimization is modified using bird swarm algorithm. The proposed work provides better convergence results compared to other metaheuristic algorithms used for feature selection. An IDS for the IoT-based healthcare systems is proposed by Saif et al. (2022). The proposed work uses a genetic algorithm, differential evaluation, and partial swarm optimization (PSO) for selecting an optimal feature subset. Afterward, the classification of intrusive traffic is done using k-nearest neighbor, and a decision tree classifier. Haddadpajouh et al. (2020) proposed a IDS for the IoT edge layer. The feature selection is performed using the bio-inspired gray wolf optimization (GWO) algorithm. A multi-kernel support vector machine is used for the detection of malicious traffic. Another work by Mafarja et al. (2020) employed a modified whale optimization algorithm for reducing the dimension in IoT-based IDS. The whale optimization algorithm is modified using transfer functions, and its performance is better than the original whale optimization algorithm. Hosseini et al. (2022) proposed a hybridized bio-inspired algorithm for detection botnets in the IoT network. They hybridized salp swarm algorithm and slime mold algorithm for feature selection of network traffic. Alweshah et al. (2022) proposed an emperor penguin colony based feature selection approach for the IoT-based IDS. The classification of intrusive traffic is performed using k-nearest neighbor (KNN) classifier.

Work by Fatani et al. (2021) proposed an aquila optimizer (AO) based feature selection approach for detecting intrusion in the IoT framework. In this paper, feature extraction is performed by convolutional neural network, and afterward, aquila optimizer determines the best features. The proposed work is evaluated against four datasets and the result shows the efficiency of proposed work against other related work. Krishna and Arunkumar (2021) proposed a GWO and PSO-based IDS for an IoT environment. The random forest classifier performs multiclass classification using the optimal features selected from hybrid PSO and GWO. Experimental result shows the effectiveness of the proposed system against other similar work using the NSL-KDD and N-BaIoT dataset. Furthermore, work by Sarwar et al. (2022) proposed a feature selection algorithm for IDS. They detect optimal features using an improved dynamic sticky binary partial swarm optimization algorithm. Classification of IoT intrusive traffic is done using random forest (RF). Dahou et al. (2022) employed a reptile search algorithm for selecting optimal features in IoT framework. The proposed work is evaluated against multiple datasets such as NSL-KDD, BoT-IoT, KDDCup-99, and CICIDS-2017. Work by Priya et al. (2020) proposed a hybridized principal component analysis and gray wolf optimization based IDS for the Internet of Medical Things. The intrusive traffic is classified using deep neural network. The hybridized bio-inspired-based IDS is proposed by Davahli et al. (2020) for IoT system. They hybridized genetic algorithm and gray wolf optimization for optimal feature selection. Further, the intrusive traffic is classified using support vector machine classifier. The AWID dataset is used for the performance evaluation of this system. A smart IDS for IoT system is proposed by Keserwani et al. (2021). They employed hybridized GWO and PSO algorithm for feature selection task. The multiclass classification is performed using random forest classifier. The proposed work is evaluated against multiple datasets.

Hence, this paper proposes hybridized IDS for the IoT system. The feature selection is done by hybridizing the bio-inspired sine cosine algorithm (SCA), and salp swarm algorithm (SSA) for selecting the optimal feature subset. The proposed approach is compared with other bio-inspired algorithms used for the FS task in the IoT-based IDS. The dataset IoTID20 is used to verify and evaluate system performance. Multiclass classification is performed using two machine learning classifiers and afterward, their performance is analyzed. The performance of hybridized SCA-SSA with KNN and XGBoost classifiers is better compared to other metaheuristic algorithms used for feature selection. The paper is formulated as: Section 2 describes the material and methods used by the hybridized IoT-based IDS, and Section 3 describes the implementation result. Section 4 presents the discussion.

## 2. Material and methods

### 2.1. Background

#### 2.1.1. Sine cosine algorithm (SCA)

This math-based algorithm is defined by Mirjalili (2016). SCA is motivated by trigonometric properties of sine and cosine functions for updating individual positions which provide an optimal solution for optimization problems. SCA is easy to implement, flexible and the probability of falling in local optima is low. However, SCA might suffer from premature convergence. The solutions are updated using the following equations:


(1) $$\begin{array}{l} X_{m,n}^{ite+1}=\;X_{m,n}^{ite}+rnd_{1}\times \sin\;(rnd_{2})\;\times \;\left|rnd_{3}\;X_{Best\;n}^{ite}-\;X_{m,n}^{ite}\right|\;\\ \;if\;rnd_{4}<0.5 \end{array}$$

(2) $$\begin{array}{l} X_{m,n}^{ite+1}=\;X_{m,n}^{ite}+rnd_{1}\;\times \;\cos\;(rnd_{2})\;\times \;\left|rnd_{3}\;X_{Best\;n}^{ite}-\;X_{m,n}^{ite}\right|\;\\ \;if\;rnd_{4}\geq 0.5 \end{array}$$

where, $X_{m,n}^{ite}$ is a current solution of individual m in *n-*th dimension at iteration *ite*. $X_{Best\;n}^{ite}$ is the best solution at iteration *ite* in the *n-*th dimension. *rnd*_1_, *rnd*_2_, *rnd*_3_,, *and rnd*_4_ are random numbers. *rnd*_2_ lies between[0, 2π]. *rnd*_3_ controls the search agent mobility direction. *rnd*_4_ is used to switch between two search methods. *rnd*_1_ controls the exploration and exploitation phase and is updated using the following equation:


(3) $$\begin{array}{l} {rnd}_{1}=k-\frac{k}{{ite}_{\max}}\times ite \end{array}$$

(4) $$\begin{array}{l} {rnd}_{2}=2\times \pi \times \;random\;number\left(0,1\right) \end{array}$$

where, k is constant, *ite*_max_ is the maximum iteration and *ite* is the current iteration.

#### 2.1.2. Salp swarm algorithm (SSA)

This algorithm is proposed by Mirjalili et al. (2017). SSA is motivated by the hunting habits of salps in the sea. They belong to salpidae family, and their movement is alike jellyfish. They form a chain while living in a group. It required a few parameter settings and is simple to implement. However, SSA might suffer from premature convergence. The foremost salp in a chain is the leader, and others are followers. Leader position is determined with the following equation:


(5) $$\begin{array}{l} X_{n}^{1}={FPos}_{j}-{crn}_{1}\left[\left(UpBn-LoBn\right){crn}_{2}+LoBn\right]\;if\;{crn}_{3}\;<\;0.5 \end{array}$$

(6) $$\begin{array}{l} X_{n}^{1}={FPos}_{j}+{crn}_{1}\left[\left(UpBn-LoBn\right){crn}_{2}+LoBn\right]\;if\;{crn}_{3}\;\geq \;0.5 \end{array}$$

where, $X_{n}^{1}$ is the leader position, *FPos*_*j*_ food position. *UpBn* is upper bound. *LoBn* is lower bound. *crn*_2_ is a random number between [0,1] used to control the mobility step of the leader. *crn*_3_ controls the switch between two position-updating equations. *crn*_1_ is the control parameter that balances SSA execution. It is defined by the following equation:


(7) $$\begin{array}{l} {crn}_{1}=2e^{-\left(\frac{4\;ite}{{ite}_{\max}}\right)} \end{array}$$

The follower position is determined using the following equation:


(8) $$\begin{array}{l} X_{n}^{m}=\frac{1}{2}\left(X_{n}^{m}+X_{n}^{m-1}\right) \end{array}$$

where $X_{n}^{m}$ represents the *m*-th follower in *n-*th dimension.

### 2.2. Proposed system

**Figure 1:**
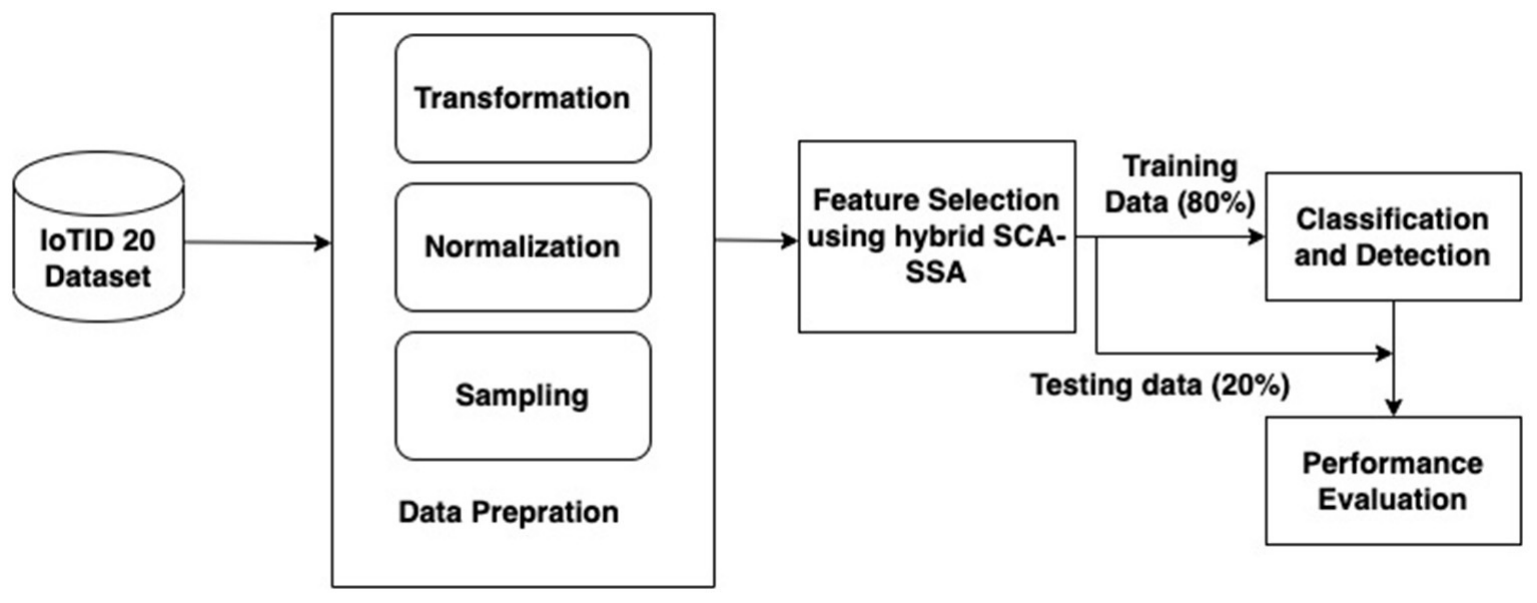
Proposed system framework.

A hybridized IDS for the IoT framework has been proposed in this section. Figure 1 depicts the proposed system and it is divided into the following parts including, data preparation, feature selection using SCA-SSA, classification, and detection, and performance evaluation.

#### 2.2.1. IoTID20 dataset

The IoTID20 (Ullah and Mahmoud, 2020) dataset is used as collected data in this paper. The IoTID20 is the result of the testbed configuration of the smart home. Smart home devices such as laptops, tablets, Wi-Fi cameras, smartphones, and other devices are used to generate network flow data for the IoTID20 dataset. These devices are divided into categories of victim devices i.e., EZVIZ Wi-Fi camera, SKT NGU, and the attacking devices including smartphones, tablets, etc. all other IoT devices. This labeled dataset includes 83 features. The five attack category instances of the IoTID20 dataset used for performance evaluation are shown in Table 1.

**Table 1:** IoTID20 dataset instances.

**Category**	**Instances**	**Category**	**Instances**	**Category**	**Instances**
Mirai	415,677	DoS	59,391	MITM ARP Spoofing	35,377
Scan	75,265	Normal	40,073		

#### 2.2.2. Data preparation

The performance of the learning algorithm depends upon the type of data provided as input. Therefore, various data preparation techniques such as transformation, normalization, and sampling are employed to improve the quality of data. A vast amount of heterogeneous data is collected from IoT devices with numerous features. However, not all features are useful for the classification task, few of them are irrelevant and redundant. Therefore, these redundant and irrelevant attributes are eliminated. Afterward, categorical feature values are transformed into numerical values using the Label encoder function. The numerical feature value of the dataset might vary. Therefore, normalization is performed so that the values lie within a range of (0, 1).

In this paper, the standard scaler function is used for this purpose. The IoTID20 dataset is unbalanced. The instances of intrusive traffic are much more than instances of normal traffic. The unbalanced dataset degrades the performance of the classifier. Therefore, random sampling is used to make the IoTID20 dataset balanced.

#### 2.2.3. Feature selection using hybrid SCA-SSA

The hybrid SCA-SSA that merges SCA with SSA is described in this section. The first part of SSA is enhanced using the SCA algorithm for updating the salp positions. During these modifications, the trigonometric functions of SCA are used to update the salp leader position. This enhances SSA flexibility for exploring search space and leads to providing optimal solutions. Afterward, the exploitation capability of salp is enhanced by adding the levy flight (LF) (Chawla and Duhan, 2018) to it. The follower position is updated using LF. LF controls the size of a step taken by salps. These enhancements lead to better convergence speed and avoid local optima. Therefore, the hybridization of SCA-SSA is shown in Algorithm 1 and is described as follows.

**Algorithm 1:** Hybrid SCA-SSA

1. Population initialization as i = 1,2,….,n 2. For each salp compute fitness function $X_{n}^{ite}=$ the best searchagent 3. While(*ite* ≤ *ite*_max_) 4. Update LF, *crn*_1_, *rnd*_2_, *crn*_4_, *rnd*_3_, and *crn*_3_ 5. If (*ite* = =1) 6. If (*crn*_3_≥0.5) 7. Use Eq. (9) to update salp position #SALP LEADER 8. else if (*crn*_3_ < 0.5) 9. Use eq. (10) to update salp position #SALP LEADER 10. else if (*ite* ≥ 2) 11. Use Eq. (11) to update salp positon #SALP FOLLOWER 12. end if 13. *ite* = *ite* +1 14. end while 15. return optimal feature subset

##### 2.2.3.1. First part

The first part of SSA is enhanced by using the trigonometric functions of SCA, and inertia weight, which improves the exploring capability of SSA. The leader position in SSA is updated the using following equation:


(9) $$\begin{array}{l} X_{n}^{1}=w\times FPos_{j}+crn_{1}\times \sin\;(rnd_{2})\times \;\left|rnd_{3}\;X_{Best\;n}^{ite}-\;X_{n}^{ite}\right|\;\\ \;if\;crn_{3}\;\geq \;0.5 \end{array}$$

(10) $$\begin{array}{l} X_{n}^{1}=w\times FPos_{j}-crn_{1}\times \cos\;(rnd_{2})\times \left|rnd_{3}\;X_{Best\;n}^{ite}-\;X_{n}^{ite}\right|\;\\ \;if\;crn_{3}<\;0.5 \end{array}$$

where, *w* = 0.9 is the inertia weight, *FPos*_*j*_ food position in *j*-th dimension. *crn*_1_ is obtained from Eq. (7). *rnd*_2_ is obtained using Eq. (4). *rnd*_3_ and *crn*_3_ are random numbers. $X_{Best\;n}^{ite}$ is best solution at iteration *ite* in *n-*th dimension, and $X_{n}^{ite}$ is current solution of individual.

##### 2.2.3.2. Second part

The second part of SSA is enhanced by introducing levy flight function. Therefore, the follower position is determined by


(11) $$\begin{array}{l} X_{n}^{m}=\frac{1}{2}\left(X_{n}^{m}+X_{n}^{m-1}\right)+0.01\times LF\times \;{crn}_{4} \end{array}$$

where, *crn*_4_ is random number between [0,1], $X_{n}^{m}$ represents the *m*-th follower in *n-*th dimension. LF function is defined by following equation:


(12) $$\begin{array}{l} LF=\;\frac{f\;\times \;\sigma }{|g{|}^{\frac{1}{\beta }}},\;\text{where }\sigma =\;{\left(\;\frac{\Gamma \left(1+\beta \right)\times \sin\left(\frac{\pi \beta }{2}\right)}{\Gamma \left(\frac{1+\beta }{2}\right)\times \;\beta \times 2^{\left(\frac{\beta -1}{2}\right)}}\right)}^{\frac{1}{\beta }} \end{array}$$

where, β = 1.25. f and g are the random numbers, between [0, 1].

#### 2.2.4. Classification and detection

With the feature subset obtained from the feature selection phase, classification and detection is performed using machine learning classifier. In this paper, multiclass classification is done i.e., detecting each attack category. In this paper, KNN and XGBoost classifier is used to identify intrusive network flow traffic. This sub-section describes these machine learning classifiers briefly.

##### 2.2.4.1. KNN

The KNN is one the simplest supervised machine learning classifier. This classification method uses statistical measures to determine the instances proximity. K represents the number of neighbors. The data instances with high similarity belongs to same class. KNN allows new instances to be labeled using the previously labeled instances. However, it is highly sensitive to noise.

##### 2.2.4.2. XGBoost

This machine learning classifier allows to create sequential decision trees. This classifier is widely used for classification and regression task. XGBoost is used to speed-up the classification task and improving the classification performance. However, this classifier doesn't work well with unstructured data.

## 3. Result

The experiment is conducted on MAC Catalina OS with 8GB RAM. The implementation of classifiers and feature selection algorithms is done using python programming language. The formula of metrics used to evaluate performance is given in Table 2.

**Table 2:** Metric formula.

**Metric**	**Formula**	**Metric**	**Formula**
Accuracy	$\frac{\left(TPS+TNS\right)}{\left(TPS+FNS+TNS+FPS\right)}$	Recall (R)	$\frac{TPS}{\left(TPS+FNS\right)}$
Precision (P)	$\frac{TPS}{\left(TPS+FPS\right)}$	F-Score	$\frac{2\times R\times P}{\left(R+P\right)}$

Where, FNS, false negative; TPS, true positive; TNS, true negative; FPS, false positive.

**Time** is the total computation time an IDS model will take with feature selection algorithm (in secs).

Number of features (N.F.) defines length of features selected by feature selection algorithm.


$$\begin{array}{l} \text{Fitness Function}=\;\propto \;\times \;\left(1-Accuracy\right)+\beta \;\\ \;\times \;\frac{N.F.\;}{Maximum\;features}\text{ where},\text{ α}=0.99,\text{ β}=1-\text{α} \end{array}$$

### 3.1. Result analysis

**Figure 2:**
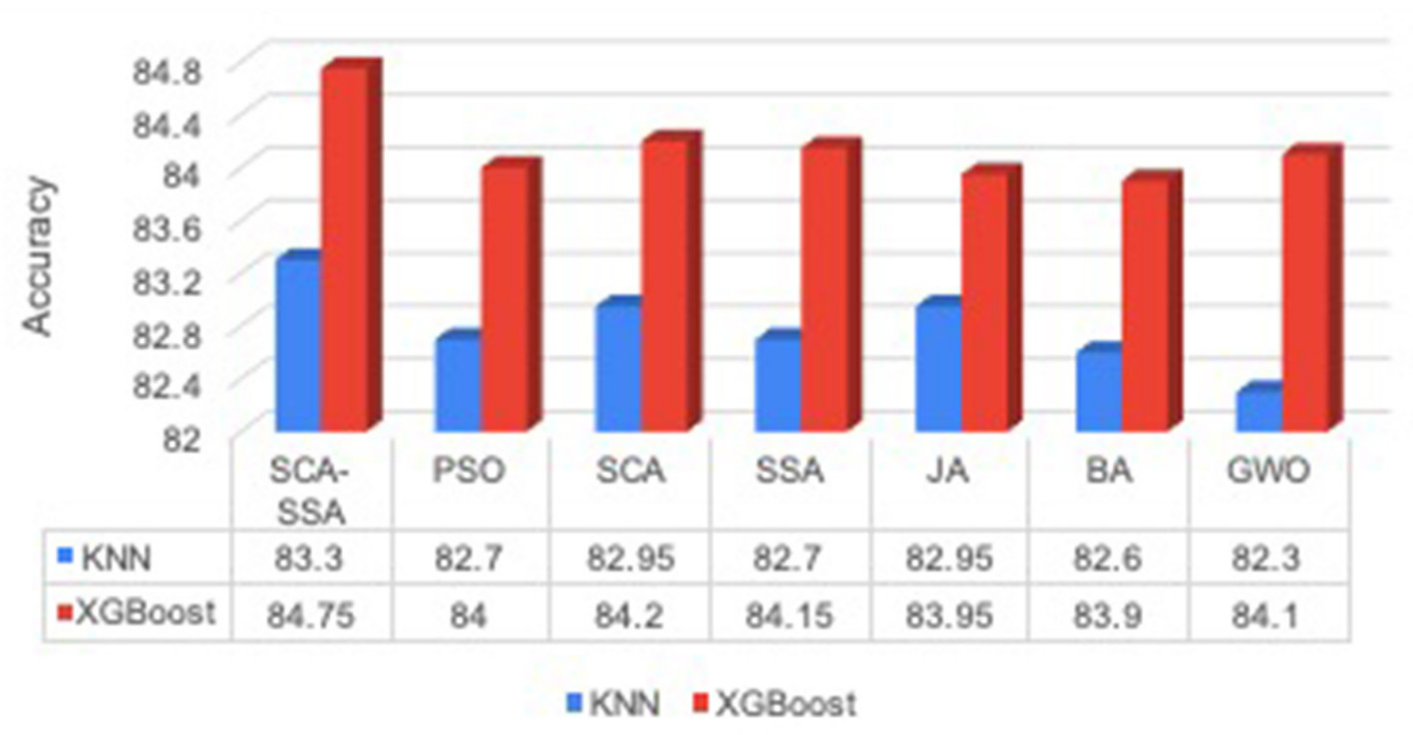
Accuracy.

The proposed system is compared with the following algorithm such as sine-cosine algorithm (SCA) (Mirjalili, 2016), salp swarm algorithm (SSA) (Mirjalili et al., 2017), Jaya Algorithm (JA) (Rao, 2016), Bat algorithm (BA) (Yang, 2010), and grey wolf optimization algorithm (GWO) (Mirjalili et al., 2014), used for the feature selection task of IDS. Afterward the performance of two classifiers KNN and XGBoost is compared with these feature selection methods. Figure 2 depicts that the SCA-SSA with KNN and XGBoost attains the highest accuracy of 83.3% with the KNN classifier and 84.75% with the XGBoost classifier. On the other side, BA attains the minimum accuracy of 82.6% with KNN and 83.9% with the XGBoost classifier.

**Figure 3:**
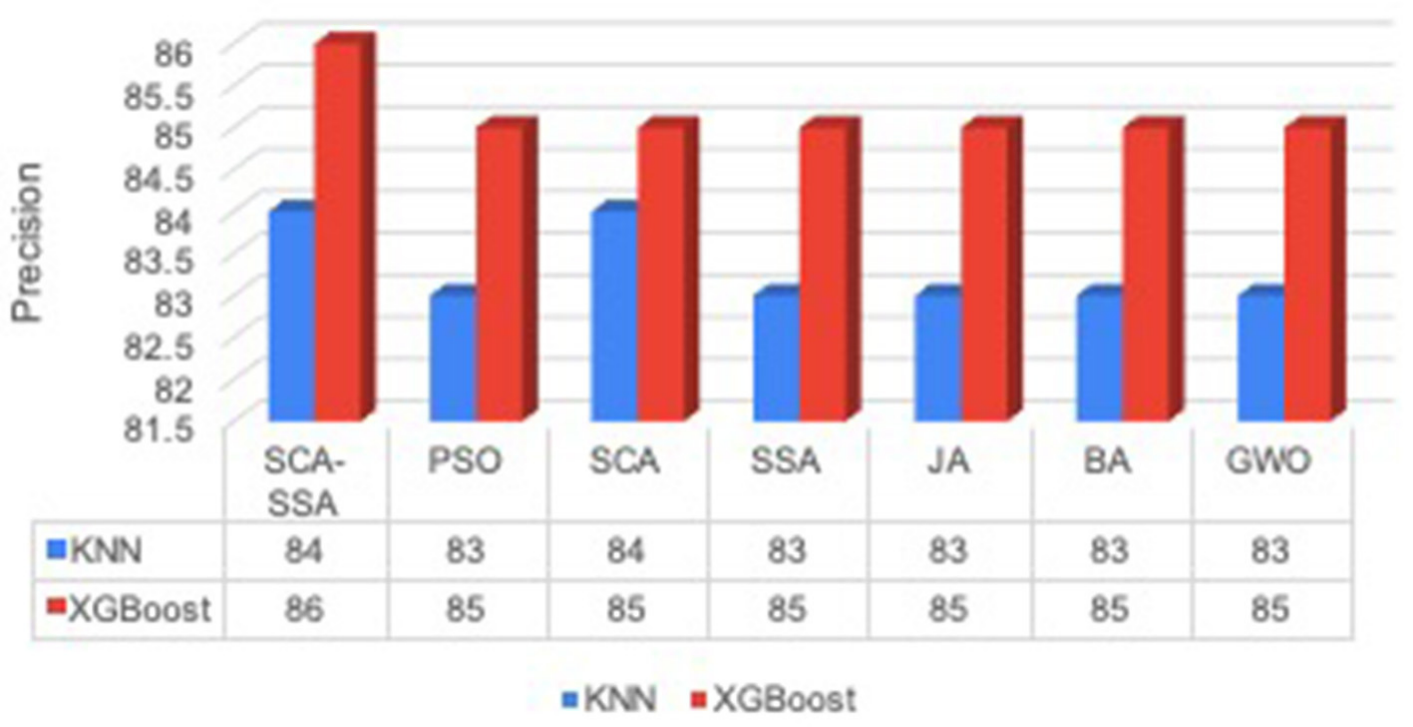
Precision.

**Figure 4:**
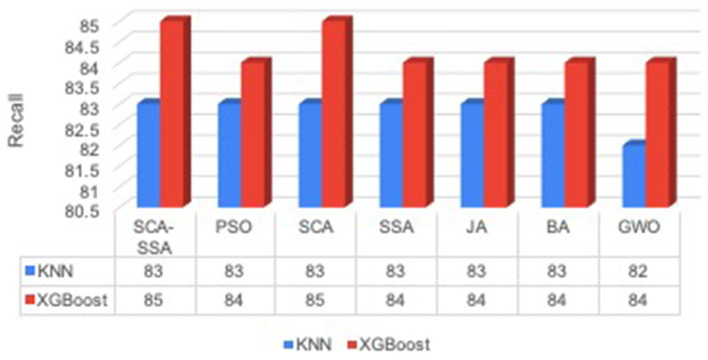
Recall.

**Figure 5:**
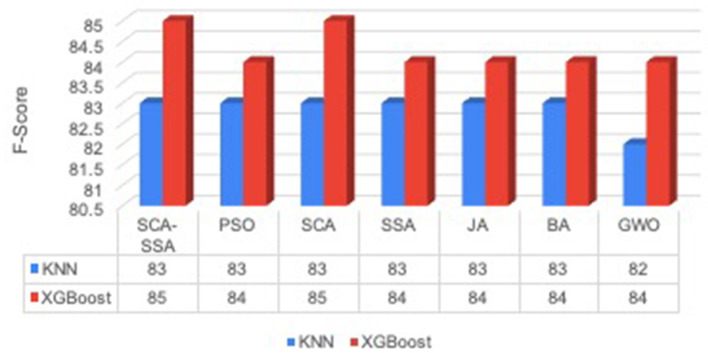
*F*-score.

**Figure 6:**
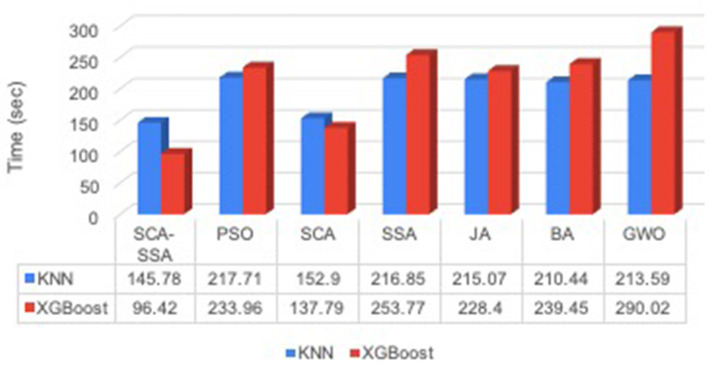
Time.

**Figure 7:**
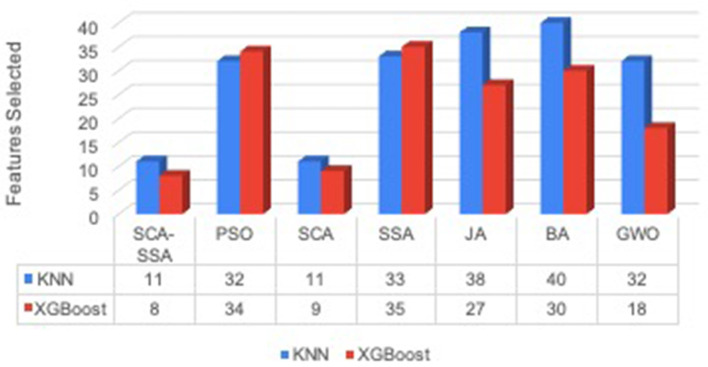
Feature selected.

**Figure 8:**
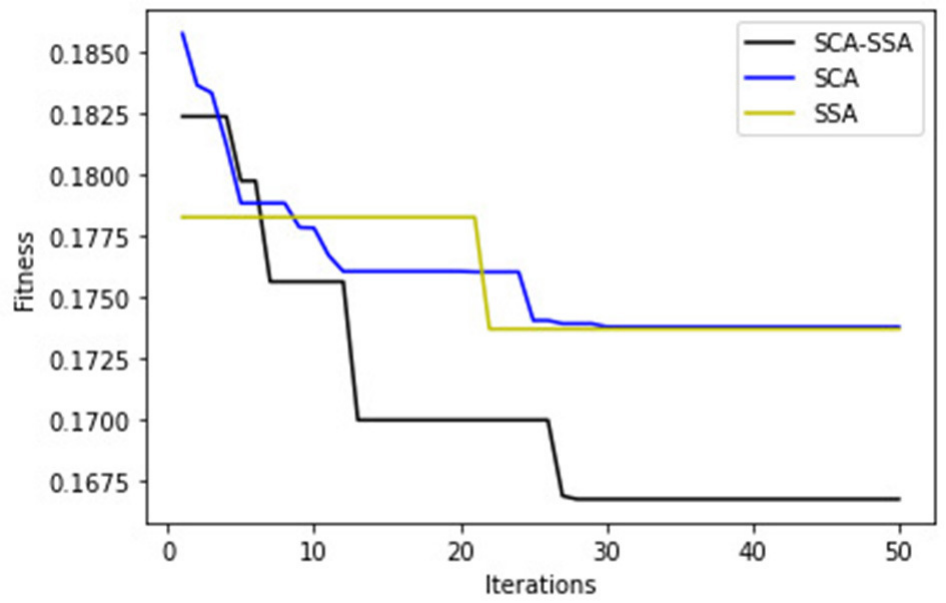
KNN-convergence curve.

**Figure 9:**
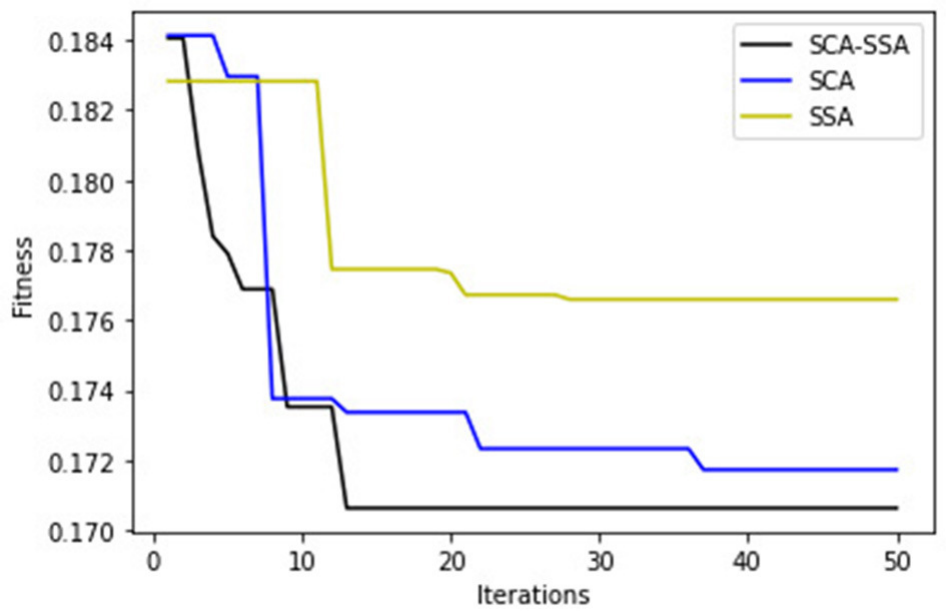
XGBoost-convergence curve.

Figure 3 depicts that SCA-SSA attains a precision of 84% with KNN and 86% with XGBoost, which is the highest among all other methods. Similarly, recall and F-Score of SCA-SSA are highest among other methods with KNN and XGBoost classifier as shown in Figures 4, 5, respectively. SCA-SSA takes the lowest execution time of 145.78 s with KNN, and 96.42 s with XGBoost as depicted in Figure 6. SSA takes the highest execution time with KNN, and GWO takes the highest execution time with the XGBoost classifier. Figure 7 shows SCA-SSA selects features with a minimal length of 11 with KNN and 8 with XGBoost classifier. Figures 8, 9 show the convergence curve of hybridized SCA-SSA with KNN, and XGBoost, respectively, which is better compared to convergence curve of original SCA, and original SSA. The overall performance of SCA-SSA with XGBoost classifier is better compared to SCA-SSA with KNN classifier in terms of all metrics used for performance evaluation of this system.

## 4. Discussion

Prevention of IoT devices from malicious activities is crucial. Data produced by these smart devices are heterogeneous. Hence, an IDS with an efficient feature selection method is important, with an objective of reducing the data size and detection time without compromising the system accuracy. The hybridized bio-inspired IDS for the IoT system is proposed in this paper. The IoTID20 dataset determines the performance of the proposed work. The optimal features are selected using hybridized SCA-SSA algorithm from pre-processed data. Then KNN and XGBoost classifiers are used to perform multiclass classification of intrusive data. Experimental result shows that the proposed system ameliorates other similar approaches. The convergence rate of SCA-SSA is better compared to SCA, and SSA. The hybrid SCA-SSA is compared against other metaheuristic algorithm used for feature selection tasks such as SCA, SSA, JA, BA, and GWO. The result shows that hybrid SCA-SSA with KNN and XGBoost attain high accuracy with the least execution time. Furthermore, the number of features selected is lowest with KNN and XGBoost for hybrid SCA-SCA. Overall, the performance of SCA-SSA-XGBoost is better compared to SCA-SSA-KNN. In the future, we can use deep learning models for better performance of the proposed work.

## Data availability statement

The original contributions presented in the study are included in the article/supplementary material, further inquiries can be directed to the corresponding author/s. The IoTID20 dataset is analyzed for this research. This dataset is publically accessible at: https://sites.google.com/view/iot-network-intrusion-dataset/home.

## Author contributions

RS done paper drafting, implementation, and paper writing under the supervision of RU. All authors contributed equally in paper review. All authors contributed to the article and approved the submitted version.
